# Permanent draft genome of *Thiobacillus thioparus* DSM 505^T^, an obligately chemolithoautotrophic member of the *Betaproteobacteria*

**DOI:** 10.1186/s40793-017-0229-3

**Published:** 2017-01-19

**Authors:** Lee P. Hutt, Marcel Huntemann, Alicia Clum, Manoj Pillay, Krishnaveni Palaniappan, Neha Varghese, Natalia Mikhailova, Dimitrios Stamatis, Tatiparthi Reddy, Chris Daum, Nicole Shapiro, Natalia Ivanova, Nikos Kyrpides, Tanja Woyke, Rich Boden

**Affiliations:** 1 School of Biological and Marine Sciences, University of Plymouth, Drake Circus, Plymouth, PL4 8AA UK; 2 Sustainable Earth Institute, University of Plymouth, Drake Circus, Plymouth, PL4 8AA UK; 3 DOE Joint Genome Institute, 94598 Walnut Creek, CA USA

**Keywords:** *Thiobacillus thioparus*, *Betaproteobacteria*, Sulfur oxidation, Chemolithoautotroph, Carboxysome, Denitrification

## Abstract

*Thiobacillus thioparus* DSM 505^T^ is one of first two isolated strains of inorganic sulfur-oxidising *Bacteria*. The original strain of *T. thioparus* was lost almost 100 years ago and the working type strain is Culture C^T^ (=DSM 505^T^ = ATCC 8158^T^) isolated by Starkey in 1934 from agricultural soil at Rutgers University, New Jersey, USA. It is an obligate chemolithoautotroph that conserves energy from the oxidation of reduced inorganic sulfur compounds using the Kelly-Trudinger pathway and uses it to fix carbon dioxide It is not capable of heterotrophic or mixotrophic growth. The strain has a genome size of 3,201,518 bp. Here we report the genome sequence, annotation and characteristics. The genome contains 3,135 protein coding and 62 RNA coding genes. Genes encoding the transaldolase variant of the Calvin-Benson-Bassham cycle were also identified and an operon encoding carboxysomes, along with Smith’s biosynthetic horseshoe *in lieu* of Krebs’ cycle *sensu stricto*. Terminal oxidases were identified, *viz*. cytochrome *c* oxidase (*cbb*3, EC 1.9.3.1) and ubiquinol oxidase (*bd*, EC 1.10.3.10). There is a partial *sox* operon of the Kelly-Friedrich pathway of inorganic sulfur-oxidation that contains *soxXYZAB* genes but lacking *soxCDEF*, there is also a lack of the DUF302 gene previously noted in the *sox* operon of other members of the ‘*Proteobacteria*’ that can use trithionate as an energy source. In spite of apparently not growing anaerobically with denitrification, the *nar*, *nir*, *nor* and *nos* operons encoding enzymes of denitrification are found in the *T. thioparus* genome, in the same arrangements as in the true denitrifier *T. denitrificans*.

## Introduction

In 1902, *Thiobacillus thioparus* was one of the first two obligately chemolithoautrophic sulfur-oxidizing *Bacteria* to be isolated, (along with what is now *Halothiobacillus neapolitanus*), and was named in 1904 [1, 2]. The original isolates were lost, but *Thiobacillus thioparus* is now the type species of the genus (a member of the *Betaproteobacteria*) and the type strain is an isolate from Starkey (1934) (= Culture C^T^ = DSM 505 ^T^ = ATCC 8158 ^T^ = CIP 104484 ^T^ = JCM 3859 ^T^ = NBRC 103402 ^T^) [3, 4]. Originally, the characteristic of utilizing inorganic sulfur compounds as an energy source was thought to be a taxonomic trait unique to *Thiobacillus* and at its height the genus contained in at least 32 different ‘species’ [4]. With phylogenetic methods however, many of these strains have since been reassigned to different, often new genera with *T. thioparus* DSM 505 ^T^ being one of four species with validly published names left in the genus. Compared to other inorganic sulfur-oxidisers, surprisingly little research has been conducted on *T. thioparus* DSM 505 ^T^ in terms of physiology and biochemistry or genetics, possibly due to the low growth yields of this species when compared to *T. denitrificans* and *T. aquaesulis* [5, 6] making it more challenging to study. It may, however, give extended and contrasted insights into autotrophic sulfur-oxidation in the *Bacteria*. It was selected for genome sequencing as part of the Department of Energy DOE-CSP 2012 initiative – as type species of a genus.

## Organism information

### Classification and features

**Fig. 1:**
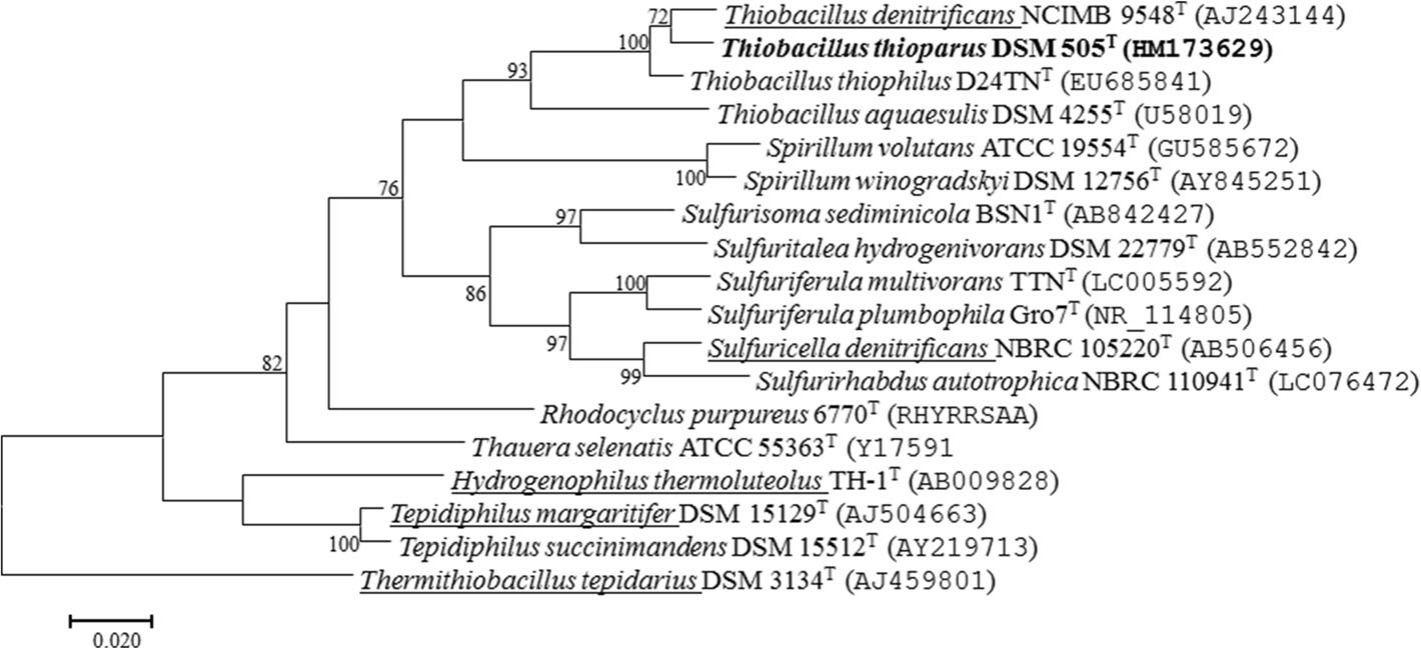
Maximum-likelyhood phylogenetic tree based on MUSCLE alignment of 16S rRNA gene sequences of the genus *Thiobacillus* and the closely related members of the *Betaproteobacteria*. Type strains of each species are used and only species with validly published names are shown. Sequences pertaining to organisms for which a publically available genome sequence exists are underlined. Accession numbers for the GenBank database are in parentheses. Alignment and tree were constructed in MEGA 6 [53]. Tree was drawn using the Tamura-Nei model for maximum-likelyhood trees [54]. Values at nodes are based on 5,000 bootstrap replicates, with values <70% omitted. Scale-bar indicates 2 substitutions per 100. *Thermithiobacillus tepidarius* DSM 3134^T^ from the *Acidithiobacillia* is used as the outgroup

*Thiobacillus thioparus* DSM 505 ^T^ was isolated from sandy loam soil from the New Jersey Agricultural Experimental Station planted with unspecified vegetable crops using 20mM thiosulfate as sole energy source in basal medium at pH 8.5 by Starkey (1934) [3] and is currently one of four species with validly published names within the genus. It forms small white colonies of 1–3 mm diameter after 2–3 days that turn pink or brown with age and which become coated with yellow-ish elementary sulfur. When grown in liquid media, finely divided (white) elementary sulfur is formed during early stages of growth, particularly when thiosulfate is used as the energy source. This disappears when growth approaches stationary phase. Thiosulfate is oxidized stoichiometrically to tetrathionate after 24 h accompanied by a rise in pH, characteristic of the Kelly-Trudinger pathway. Tetrathionate is subsequently oxidized to sulfate with culture pH falling to pH 4.8 by stationary phase. During continuous culture, no intermediates are detected in the medium once steady-state has been established. If the dilution rate of a thiosulfate limited chemostat is increased, a large production of elementary sulfur is observed, which disappears as a new steady-state is established at the faster dilution rate. General features of *T. thioparus* DSM 505 ^T^ are summarized in Table 1. A phylogenetic tree based on the 16S rRNA gene sequence showing this organisms position within the *Betaproteobacteria* and rooted with *Thermithiobacillus tepidarius* is given in Fig. 1.

**Table 1:** Classification and general features of Thiobacillus thioparus DSM 505^T^ according to MIGS recommendations [43]

MIGS ID	Property	Term	Evidence code^a^
	Classification	Domain *Bacteria*	TAS [44]
		Phylum ‘*Proteobacteria*’	TAS [45]
		Class *Betaproteobacteria*	TAS [46]
		Order *Hydrogenophilales*	TAS [47]
		Family *Hydrogenophilaceae*	TAS [48]
		Genus *Thiobacillus*	TAS [2, 3]
		Species *Thiobacillus thioparus*	TAS [2, 3]
		(Type) strain: DSM 505^T^	TAS [49]
	Gram stain	*Negative*	TAS [3]
	Cell shape	*Rod*	TAS [2, 3]
	Motility	*Motile*	TAS [2, 3]
	Sporulation	*None*	TAS [3]
	Temperature range	*N.D.*	NAS
	Optimum temperature	*28 °C*	TAS [3, 50]
	pH range; Optimum	*N.D.; 7.0*	NAS
	Carbon source	*Carbon dioxide*	TAS [3, 50]
MIGS-6	Habitat	*Agricultural soil (sandy loam)*	TAS [3]
MIGS-6.3	Salinity	*N.D.*	NAS
MIGS-22	Oxygen requirement	*Aerobic*	TAS [3, 50]
MIGS-15	Biotic relationship	*Free-living*	TAS [3, 50]
MIGS-14	Pathogenicity	*Non-pathogen*	NAS
MIGS-4	Geographic location	*New Jersey, United States of America*	TAS [3]
MIGS-5	Sample collection	*1934*	TAS [3]
MIGS-4.1	Latitude	*40° 28′ 57″ N*	TAS [3]
MIGS-4.2	Longitude	*74° 26′ 14″ E*	TAS [3]
MIGS-4.4	Altitude	*28 m*	TAS [3]

^a^ Evidence codes - *IDA* Inferred from Direct Assay, *TAS* Traceable Author Statement (i.e., a direct report exists in the literature), *NAS* Non-traceable Author Statement (i.e., not directly observed for the living, isolated sample, but based on a generally accepted property for the species, or anecdotal evidence). These evidence codes are from the Gene Ontology project [51, 52]

**Fig. 2:**
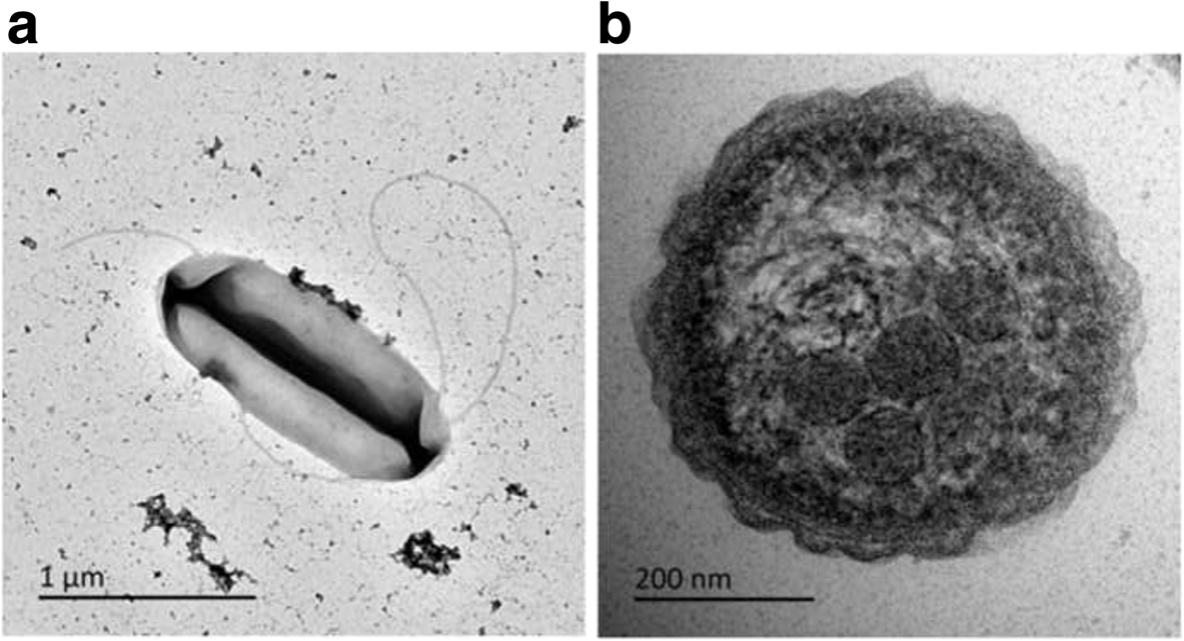
Transmission electron micrographs of *T. thioparus* DSM 505^T^ cells obtained from a thiosulfate-limited chemostat (20 mM, *D* = 0.07 h^−1^) visualized in a JEOL JEM-1400Plus transmission electron microscope, operating at 120 kV. **a** Negatively stained cells. Cells were applied to Formvar® and carbon coated copper grid before washing with saline and staining in 50 mM uranyl acetate for 5 mins and washing again. **b** Sectioned cells showing the presence of an electron dense polyphosphate (‘volutin’) granule and numerous polyhedral carboxysomes that are paler in comparison

Cells are 1.5–2.0 by 0.6 to 0.8 μm and stain Gram negative. They are motile by means of a single polar flagellum between 6 and 10 μm in length, as shown in Fig. 2. The dominant respiratory quinone is ubiquione-8 [7–10] and fix carbon dioxide using the Calvin-Benson-Bassham cycle at the expense of inorganic sulfur oxidation. Cells accumulate polyphosphate (‘volutin’) granules in batch culture and to a lesser extent in an energy-limited chemostat [8]. Carboxysomes are present in cells regardless of growth conditions employed (Fig. 1), but are apparently not found in *T. denitrificans* cells, at least not under growth conditions employed in previous studies [9]. *T. thioparus* can only use oxygen as a terminal electron acceptor – nitrate, nitrite, thiosulfate, sulfate, elementary sulfur and ferric iron do not support growth as terminal electron acceptors. The genomic DNA G + C content has been estimated using the thermal denaturation [11] at 61–62 mol% [7, 10]. *T. thioparus* DSM 505 ^T^ does not grow on any organic carbon compound tested, including sugars (glucose, ribose, fructose, sucrose), intermediates of Krebs’ cycle (citrate, succinate, fumarate, malate, oxaloacetate), carboxylates (glycolate, formate, acetate, propionate, pyruvate), one-carbon compounds (monomethylamine, dimethylamine, trimethylamine, methanol, methane), structural amino acids (all 20), substituted thiophenes (thiophene-2-carboxylate, thiophene-3-carboxylate) or complex media (yeast extract, nutrient broth, brain-heart infusion, Columbia sheep or horse blood agar, chocolate agar). Energy sources that support autotrophic growth of DSM 505 ^T^ include thiosulfate, trithionate, tetrathionate, pentathionate, hexathionate, thiocyanate and dithionate. Some *bone fide* strains of *T. thioparus* (Tk-m and E6) grow autotrophically on carbon disulfide, dimethylsulfide, dimethyldisulfide and Admidate (*O,O*-dimethylphosphoramidothioate) [4], but it is not known if the type strain DSM 505 ^T^ is capable of this. Autotrophic growth is not supported by Fe(II), Mn(II), Cu(I), U(IV), sulfite, dimethylsulfoxide, dimethylsulfone, pyrite or formate. During batch growth on thiosulfate the intermediate production of tetrathionate is observed during early stages of growth, indicative of the Kelly-Trudinger pathway [12]. Compared to the other two members of the genus [5, 6], *T. thioparus* DSM 505 ^T^ has relatively low growth yields on thiosulfate (Hutt and Boden, *manuscript in preparation*) which may give insight into the physiological variances of Kelly-Trudinger pathway organisms even within one genus.

## Genome sequencing information

### Genome project history

This organism was selected for sequencing on the basis of its role in sulfur cycling, physiological, biochemical, evolutionary and biogeochemical importance, and is part of the GEBA-KMG project at the U.S. Department of Energy JGI. The genome project is deposited in the Genomes OnLine Database [13] and a high-quality permanent draft genome sequence in IMG (the annotated genome is publically available in IMG under Genome ID 2515154076) [14]. Sequencing, finishing and annotation were performed by the JGI using state of the art sequencing technology [15]. A summary of the project information is given in Table 2.

**Table 2:** Project information

MIGS ID	Property	Term
MIGS 31	Finishing quality	Improved High-Quality Draft
MIGS-28	Libraries used	Illumina Standard PE
MIGS 29	Sequencing platforms	Illumina
MIGS 31.2	Fold coverage	122.7
MIGS 30	Assemblers	Allpaths/Velvet
MIGS 32	Gene calling method	NCBI Prokaryotic Genome Annotation Pipeline
	Locus Tag	B058
	Genbank ID	ARDU00000000
	GenBank Date of Release	April 16th, 2013
	GOLD ID	Ga0025551
	BIOPROJECT	PRJNA169730
MIGS 13	Source Material Identifier	DSM 505^T^
	Project relevance	GEBA-KMG

### Growth conditions and genomic DNA preparation

*T. thioparus* DSM 505 ^T^ DNA was obtained from Dr Hans-Peter Klenk at the DSMZ, having been grown on basal salts medium pH 6.6, supplemented with 40 mM thiosulfate as the sole energy source (DSM Medium 36), under air at 26 °C for 72 h. DNA was extracted using the JETFLEX Genomic DNA Purification Kit from Genomed (Löhne, Germany) into TE Buffer. Quality was checked by agarose gel electrophoresis.

### Genome sequencing and assembly

The draft genome of *Thiobacillus thioparus* DSM 505 ^T^ was generated at the DOE JGI using the Illumina technology [16]. An Illumina standard shotgun library was constructed and sequenced using the Illumina HiSeq 2000 platform which generated 11,161,382 reads totalling 1,674.2 Mbp. All general aspects of library construction and sequencing performed at the JGI can be found at http://www.jgi.doe.gov. All raw Illumina sequence data was passed through DUK, a filtering program developed at JGI, which removes known Illumina sequencing and library preparation artifacts (Mingkun L, Copeland A, Han J, Unpublished). Following steps were then performed for assembly: (1) filtered Illumina reads were assembled using Velvet (version 1.1.04) (Mingkun, L., Unpublished), (2) 1–3 Kbp simulated paired end reads were created from Velvet contigs using wgsim [17], (3) Illumina reads were assembled with simulated read pairs using Allpaths–LG (version r41043) [18]. Parameters for assembly steps were: 1) Velvet (velveth: 63 –shortPaired and velvetg: −very clean yes –export-Filtered yes –min contig lgth 500 –scaffolding no –cov cutoff 10) 2) wgsim (−e 0–1 100–2 100 –r 0 –R 0 –X 0) 3) Allpaths–LG (PrepareAllpathsInputs: PHRED 64 = 1 PLOIDY = 1 FRAG COVERAGE = 125 JUMP COVERAGE = 25 LONG JUMP COV = 50, RunAllpathsLG: THREADS = 8 RUN = std shredpairs TARGETS = standard VAPI WARN ONLY = True OVERWRITE = OVERWRITE = True). The final draft assembly contained 20 contigs in 20 scaffolds. The total size of the genome is 3.2 Mbp and the final assembly is based on 392.6 Mbp of Illumina data, which provides an average 122.7× coverage of the genome.

### Genome annotation

Genes were identified using Prodigal [19], followed by a round of manual curation using GenePRIMP [20] for finished genomes and Draft genomes in fewer than 10 scaffolds. The predicted CDSs were translated and used to search the NCBI nonredundant database, UniProt, TIGRFam, Pfam, KEGG, COG, and InterPro databases. The tRNAScanSE tool [21] was used to find tRNA genes, whereas ribosomal RNA genes were found by searches against models of the ribosomal RNA genes built from SILVA [22]. Other non–coding RNAs such as the RNA components of the protein secretion complex and the RNase P were identified by searching the genome for the corresponding Rfam profiles using INFERNAL [23]. Additional gene prediction analysis and manual functional annotation was performed within the IMG platform [24] developed by the JGI, Walnut Creek, CA, USA [25, 26].

## Genome properties

The genome of *T. thioparus* DSM 505 ^T^ is 3,201,518 bp-long with a 62.3 mol% G + C content (Table 3). Of the 3,197 predicted genes, 3,135 were protein-coding genes and 62 were RNA genes. A total of 2,597 genes (81.23%) have predicted function. A total of 538 (16.83%) were identified as pseudogenes – the remainder annotated as hypothetical proteins. The properties and the statistics of the genome are given in Table 3, the distribution of genes into COG functional categories is given in Table 4. The genome is the second largest genome of obligate chemolithoautotrophs sequenced to date and is 89% (bp/bp) or 90% (protein coding genes/protein coding genes) of the size of that of *T. denitrificans* DSM 12475 ^T^ [12].

**Table 3:** Genome statistics

Attribute	Value	% of Total
Genome size (bp)	3,201,518	100.00
DNA coding (bp)	2,937,381	91.75
DNA G + C (bp)	1,994,510	62.30
DNA scaffolds	18	100.00
Total genes	3,197	100.00
Protein coding genes	3,135	98.06
RNA genes	62	1.94
Pseudo genes	538	16.83
Genes in internal clusters	267	8.35
Genes with function prediction	2,597	81.23
Genes assigned to COGs	2,258	70.63
Genes with Pfam domains	2,700	84.45
Genes with signal peptides	376	11.76
Genes with transmembrane helices	767	23.99
CRISPR repeats	2	0.04

**Table 4:** Number of genes associated with general COG functional categories

Code	Value	%age	Description
J	205	8.2	Translation, ribosomal structure and biogenesis
A	1	0.0	RNA processing and modification
K	120	4.8	Transcription
L	83	3.3	Replication, recombination and repair
B	1	0.0	Chromatin structure and dynamics
D	38	1.5	Cell cycle control, Cell division, chromosome partitioning
V	61	2.4	Defense mechanisms
T	156	6.2	Signal transduction mechanisms
M	225	9.0	Cell wall/membrane biogenesis
N	101	4.0	Cell motility
U	56	2.2	Intracellular trafficking and secretion
O	144	5.8	Posttranslational modification, protein turnover, chaperones
C	207	8.3	Energy production and conversion
G	86	3.4	Carbohydrate transport and metabolism
E	153	6.1	Amino acid transport and metabolism
F	63	2.5	Nucleotide transport and metabolism
H	144	5.8	Coenzyme transport and metabolism
I	76	3.0	Lipid transport and metabolism
P	206	8.2	Inorganic ion transport and metabolism
Q	37	1.5	Secondary metabolites biosynthesis, transport and catabolism
R	165	6.6	General function prediction only
S	143	5.7	Function unknown
-	939	29.4	Not in COGs

The total is based on the total number of protein coding genes in the genome

## Insights from the genome sequence

As an obligate autotroph, it would be expected for a complete Calvin-Benson-Bassham cycle and, *in lieu* of Krebs’ cycle, Smith’s biosynthetic horseshoe [12, 27–29]. Smith’s horseshoe representing a very near-complete Krebs’ cycle was identified, in which citrate synthase (EC 2.3.3.16), aconitase (EC 4.2.1.3), isocitrate dehydrogenase (NADP^+^, EC 1.1.1.42), succinyl coenzyme A synthase (ADP-forming, EC 2.6.1.5), succinate dehydrogenase (EC 1.3.5.1) and malate dehydrogenase (oxaloacetate decarboxylating, NADP+, EC 1.1.1.40) genes are present, but fumarase (EC 4.2.1.2) and the E3 subunit of *α*-ketoglutarate dehydrogenase (NAD+, EC 1.2.4.2) genes are absent – E1 and E2 are present. This is a comparatively large version of Smith’s horseshoe [29], which varies between Classes of the ‘*Proteobacteria*’, for example, genes for fumarase, succinate dehydrogenase and the E1 subunit of *α*-ketoglutarate dehydrogenase were recently found to be missing from *Thermithiobacillus tepidarius* DSM 3134 ^T^ [12, 30, 31] of the *Acidithiobacillia*. Activities of Krebs’ cycle enzymes assayed by Smith et al. [29] in cell-free extracts of *T. thioparus* found activities of all enzymes except for *α*-ketoglutarate dehydrogenase, indicating the E1 and E2 subunits alone were not sufficient for activity. Interestingly, Smith detected fumarase activity; however, this strain of *T. thioparus* was isolated by the authors themselves and was not DSM 505 ^T^, so there may be further variation of Smith’s horseshoe at strain level. With many species having been erroneously classified as strains of *T. thioparus* in the past that have subsequently been proven to belong to other genera [4] it is also plausible that Smith’s strain was from another specie, genus or even Class. The E1, E2 and E3 subunit genes for *α*-ketoglutarate dehydrogenase were identified in the genome of *T. denitrificans* ATCC 25259 ^T^ [32], though enzyme activity was also absent in this strain [29, 33]. It is important to stress that the full suite of enzymes of Krebs’ cycle or Smith’s horseshoe have never been assayed in *T. thioparus* DSM 505 ^T^ - clearly this is needed in order to identify if genomic data reflect true activities in vivo [27].

A complete Calvin-Benson-Bassham cycle is present, with a single copy of the large (*cbbL*) and small (*cbbS*) form I RuBisCO (EC 4.1.1.39) subunits and, owing to the presence of a transaldolase (EC 2.2.1.2) and absence of a sedoheptulose-1,7-bisphosphatase (EC 3.1.3.37) gene, we can conclude that it uses the transaldolase-variant Calvin-Benson-Bassham cycle [34]. Adjacent to these genes are *cbbO* and *cbbQ*, consistent with form IAq RuBisCO [35], which is canonically cytoplasmic, rather than carboxysomal. A cluster of 11 genes that encode carboxysome shell proteins and a carboxysome carbonic anhydrase were also identified. This cluster is located on the forward strand while on the reverse strand the RuBisCO cluster is located *c*.9 kb upstream, between which is a divergently transcribed transcriptional regulator (*cbbR*) [32]. Further evidence of carboxysome expression can be seen in transmission electron micrographs (Fig. 1). The carboxysome gene cluster (experimental data would be required to demonstrate if it is an operon or not) in *T. thioparus* DSM 505 ^T^ does not start with *cbbLS*, (as would be found in *Halothiobacillus*, *Acidithiobacillus* and *Thermithiobacillus* spp. [36]) - the lack of these genes has also been observed in *T. denitrificans* ATCC 25259 ^T^ [32] and may be a diagnostic property of this genus.

Whilst *T. thioparus* DSM 505 ^T^ cannot grow anaerobically with denitrification but *T. denitrificans* does, putative operons encoding the nitrate reductase (*nar*), nitrite reductase (*nir*), nitric oxide reductase (*nor*) and nitrous oxide reductase (*nos*) proteins of canonical denitrification are found in both *T. denitrificans* and *T. thioparus* – this may indicate a potential for the latter to grow with denitrification albeit not under conditions previously employed – for example, it may occur only under micro-oxic conditions rather than fully anoxic conditions, alternatively, some unknown factor may prevent detection of nitrate and/or the expression of these genes. No evidence for any alternative denitrification pathways [37] was found

With regard to respiration, five cytochromes *c*_553_, one cytochrome *c*_556_ and three cytochrome *b* were present. The two high-affinity terminal oxidases were both found – with multiple gene copies of cytochrome *c* oxidase (*cbb*_3_, EC 1.9.3.1) and a single copy of cytochrome *bd*-type quinol oxidase (EC 1.10.3.10). It is worth noting that *T. denitrificans* also possess the *aa*_3_ variant of cytochrome *c* oxidase (EC 1.9.3.1), which may permit it greater metabolic diversity, for example, growth under more variable oxygen partial pressures - this could potentially explain why *T. thioparus* cannot denitrify under anoxic conditions, even though it possesses the operons encoding enzymes of denitrification. It is known that microaerophilic organisms usually employ *cbb*_3_ or bd-type high-affinity terminal oxidases [37] and this may further evidence that *T. thioparus* and *T. denitrificans* may grow under such conditions, potentially with denitrification, though we cannot find any studies that demonstrate this in vivo thus far.

### Extended insights

Enzymes of the Kelly-Trudinger pathway remain poorly understood and many of the genes are yet to be identified [12]. The oxidation of thiosulfate to tetrathionate takes place *via* a cytochrome *c*-linked thiosulfate dehydrogenase (EC 1.8.2.2), one gene (*tsdA*) for which was identified in *Allochromatium vinosum*, a member of the *Gammaproteobacteria* [38]. However, *tsdA* is not present in *T. thioparus* DSM 505 ^T^ supporting the hypothesis that more than one thiosulfate dehydrogenase is present in the ‘*Proteobacteria*’ or could vary at Class level. Numerous Kelly-Friedrich pathway genes (*soxXYZAB*) were present but the remaining of the conserved *soxTRS*-*VW-XYZABCDEFGH* genes being absent [39–41]. In *Paracoccus* spp. (*Alphaproteobacteria*) *soxYZ* encodes a protein complex that binds thiosulfate *via* a cysteine residue in the initial stage of the Kelly-Friedrich pathway while *soxXA* encode cytochromes *c*_551_ and *c*_552.5_ which capture two electrons from thiosulfate oxidation. Finally *soxB* encodes a hydrolase that removes the terminal sulfone group as sulfate. Missing the SoxCD or sulfur dehydrogenase protein from the multi-enzyme system would leave a sulfur atom attached to the SoxYZ residue, preventing the action of SoxB to liberate this residue to act further with another thiosulfate molecule. An unidentified protein may participate in the release of these sulfur atoms and potentially may explain the deposits of sulfur seen during initial stages of growth. This does not explain the production of tetrathionate which still would require the activity of thiosulfate dehydrogenase. If *soxXYZAB* genes are being expressed then they may be required as a functional part of the Kelly-Trudinger pathway. The action of Sox proteins (if any) in *T. thioparus* DSM 505 ^T^ in conjunction and potentially collaboration with additional Kelly-Trudinger pathway proteins would undoubtedly be essential in resolving its chemolithoautotrophic metabolism, which remains poorly understood. An unidentified gene encoding a putative DUF302-family protein is present between *soxA* and *soxB* genes of *Thermithiobacillus tepidarius* DSM 3134 ^T^, *Acidithiobacillus thiooxidans* ATCC 19377 ^T^ and *Acidithiobacillus caldus* ATCC 51756 ^T^, and *Thiohalorhabdus denitrificans* DSM 15699 ^T^, the function of which may be important in the Kelly-Trudinger pathway [12]; however, DUF302 is not present on the *sox* operon of *T. thioparus* DSM 505 ^T^, although six unidentified genes annotated as DUF302 family proteins are present elsewhere in the genome. The *soxEF* genes encode and a flavocytochrome *c* sulfide dehydrogenase (EC 1.8.2.3) and are both found in the *T. denitrificans* genome, separate from the main *sox* gene cluster, but they are not found in *T. thioparus*, whereas dissimilatory sulfite reductase (*dsr*) genes are found in both genomes*,* as are adenylyl sulfate reductase (*aprAB*, EC 1.8.99.2) genes, the presence of which has also been confirmed in *T. aquaesulis* [42].

It was previously noted that 5.9% of the genome (178 genes) of *Thermithiobacillus tepidarius* DSM 3134 ^T^ were potential horizontally transferred genes from *Thiobacillus thioparus*, *Thiobacillus denitrificans* and *Sulfuricella denitrificans* of the *Betaproteobacteria* [12]; 96 of these genes came from the two *Thiobacillus* spp. However, very little gene transfer has taken place from members of *Acidithiobacillia* to *T. thioparus* DSM 505 ^T^ with only 6 genes from *Ttb. tepidarius* DSM 3134 ^T^, 4 from *Acidithiobacillus* spp. This is perhaps not surprising due to the thermophilic and acidophilic nature of these three *Acidithiobacillia* compared to the mesophilic requirements of *T. thioparus* and the unlikelihood of these species co-inhabiting the same environments. A far larger portion (143 genes; 4.82%) of genes were attributed to transfer from members of *Gammaproteobacteria*, many of which grow at more neutral pH and mesophilic temperatures. There was no distinct pattern of any particular metabolism pathways or resistances etc. being encoded by these potentially transferred genes.

## Conclusions

The genome of *Thiobacillus thioparus* DSM 505 ^T^ gives insights into many aspects of its physiology, biochemistry and evolution. This organism uses the transaldolase variant of the Calvin-Benson-Bassham cycle and produces carboxysomes for carbon dioxide fixation, evident from both the genome and transmission electron microscopy. The expression of both carboxysomes and RuBisCO may be regulated by the same divergently transcribed transcriptional regulator. Smith’s biosynthetic horseshoe is present *in lieu* of Krebs’ cycle *sensu stricto*, but this is unusually large as only 2 genes are missing, though *T. denitrificans* [32] is only missing 1 – this may in part explain the heterotrophic growth of *T. aquaesulis* since just one additional gene would convert Smith’s horseshoe into a functional version of Krebs’ cycle [6]. Many inorganic sulfur-oxidation genes of the *sox* cluster were found but *soxC, soxD*, *soxE* and *soxF* are absent. The *tsdA* gene for a thiosulfate dehydrogenase identified in *Allochromatium vinosum* is absent and confirmation of the presence of a different thiosulfate dehydrogenase enzyme and gene will require further study. The genome sequence will enable evolutionary studies into the nature of *Thiobacillus* and chemolithoautotrophs in general, in particular reigniting the obligate *versus* facultative and the autotrophic *versus* mixotrophic debates that have been largely absent from the literature in recent years, but genome sequences becoming available will now answer many questions proposed over 10 years ago [27, 29].

## Abbreviations

ATCC: American type culture collection
BLAST: Basic linear alignment search tool
CIP: Collection de L’institut Pasteur
COG: Clusters of orthologous groups
DSMZ: Leibniz-Institut DSMZ - Deutsche Sammlung von Mikroorganismen und Zellkulturen GmbH
GEBA: Genomic encyclopedia of *Bacteria* and *Archaea*
IMG: Integrated microbial genomes database
JCM: Japan collection of microorganisms
JGI: Joint Genome Institute
KMG: 1,000 microbial genomes project
NBRC: NITE biological resource center
NCBI: National center for biotechnology information
RuBisCO: Ribulose-1,5-bisphosphate carboxylase/oxygenase
